# Targeting Ras-binding domain of ELMO1 by computational nanobody design

**DOI:** 10.1038/s42003-023-04657-w

**Published:** 2023-03-17

**Authors:** Chunlai Tam, Mutsuko Kukimoto-Niino, Yukako Miyata-Yabuki, Kengo Tsuda, Chiemi Mishima-Tsumagari, Kentaro Ihara, Mio Inoue, Mayumi Yonemochi, Kazuharu Hanada, Takehisa Matsumoto, Mikako Shirouzu, Kam Y. J. Zhang

**Affiliations:** 1 Laboratory for Structural Bioinformatics, Center for Biosystems Dynamics Research, RIKEN, 1-7-22 Suehiro, Tsurumi, Yokohama, Kanagawa 230-0045 Japan; 2 Department of Computational Biology and Medical Sciences, Graduate School of Frontier Sciences, The University of Tokyo, Kashiwa, Chiba, 277-8561 Japan; 3 Laboratory for Protein Functional and Structural Biology, Center for Biosystems Dynamics Research, RIKEN, 1-7-22 Suehiro, Tsurumi, Yokohama, Kanagawa 230-0045 Japan; 4 Drug Discovery Structural Biology Platform Unit, Center for Biosystems Dynamics Research, RIKEN, 1-7-22 Suehiro, Tsurumi, Yokohama, Kanagawa 230-0045 Japan

**Keywords:** Protein design, X-ray crystallography

## Abstract

The control of cell movement through manipulation of cytoskeletal structure has therapeutic prospects notably in the development of novel anti-metastatic drugs. In this study, we determine the structure of Ras-binding domain (RBD) of ELMO1, a protein involved in cytoskeletal regulation, both alone and in complex with the activator RhoG and verify its targetability through computational nanobody design. Using our dock-and-design approach optimized with native-like initial pose selection, we obtain Nb01, a detectable binder from scratch in the first-round design. An affinity maturation step guided by structure-activity relationship at the interface generates 23 Nb01 sequence variants and 17 of them show enhanced binding to ELMO1-RBD and are modeled to form major spatial overlaps with RhoG. The best binder, Nb29, inhibited ELMO1-RBD/RhoG interaction. Molecular dynamics simulation of the flexibility of CDR2 and CDR3 of Nb29 reveal the design of stabilizing mutations at the CDR-framework junctions potentially confers the affinity enhancement.

## Abstract (web summary)

Structural analysis of the Ras-binding domain (RBD) of ELMO1 alone and in complex with the activator RhoG enables rational engineering and validation of a nanobody targeting ELMO1 RBD.

## Introduction

Targeting actin dynamics is of high therapeutic interest because it enables artificial control of cytoskeletal remodeling and therefore the cellular processes that require changes of cell morphology such as cell division^1^, chemotaxis^2^, autophagy^3^ and apoptosis^4^. Small GTPases Ras, Rho and Rac and their crosstalk are central hubs that regulate the equilibrium of actin assembly and disassembly^5^. More recently, the scaffold protein engulfment and cell motility protein 1 (ELMO1) was discovered as an effector of RhoG to activate Rac1 and the downstream actin polymerization^6,7^. ELMO1 cooperates with the guanine nucleotide exchange factor DOCK180^8,9^. RhoG activates Rac signaling during cell migration^6,7^ and engulfment^10^ via ELMO1/DOCK180 complex. The current model suggests the GTP-bound RhoG recruits the ELMO1 N-terminal RBD to the plasma membrane, while the C-terminal PH domain stabilizes binding of DOCK DHR-2 domain to Rac1^11,12^. This model of Rac1 activation through ELMO1/DOCK axis unveiled novel interfaces, such as PH/DHR-2 and ELMO1-RBD/RhoG, that are potentially explorable in drugging actin assembly.

In addition to RhoG, Arl4A, an Arf family GTPase, has been identified as an upstream component that interacts with ELMO1 to remodel the actin cytoskeleton^13^. BAI1, an adhesion-type GPCR, forms a complex with ELMO1/DOCK180 and promotes engulfment of apoptotic cells^14^. Interaction between Gαi2 and ELMO mediates chemotaxis of breast cancer cells^15^. Gβγ also interacts with ELMO1 to activate Rac signaling during chemotaxis^16^. Recently, the binding mode of RhoG^12^ and the BAI1 C-terminal peptide^17^ was elucidated for ELMO2, a closely related isoform of ELMO1. However, the structural basis for the interaction between ELMO1 and its upstream regulators remained unknown.

**Fig. 1:**
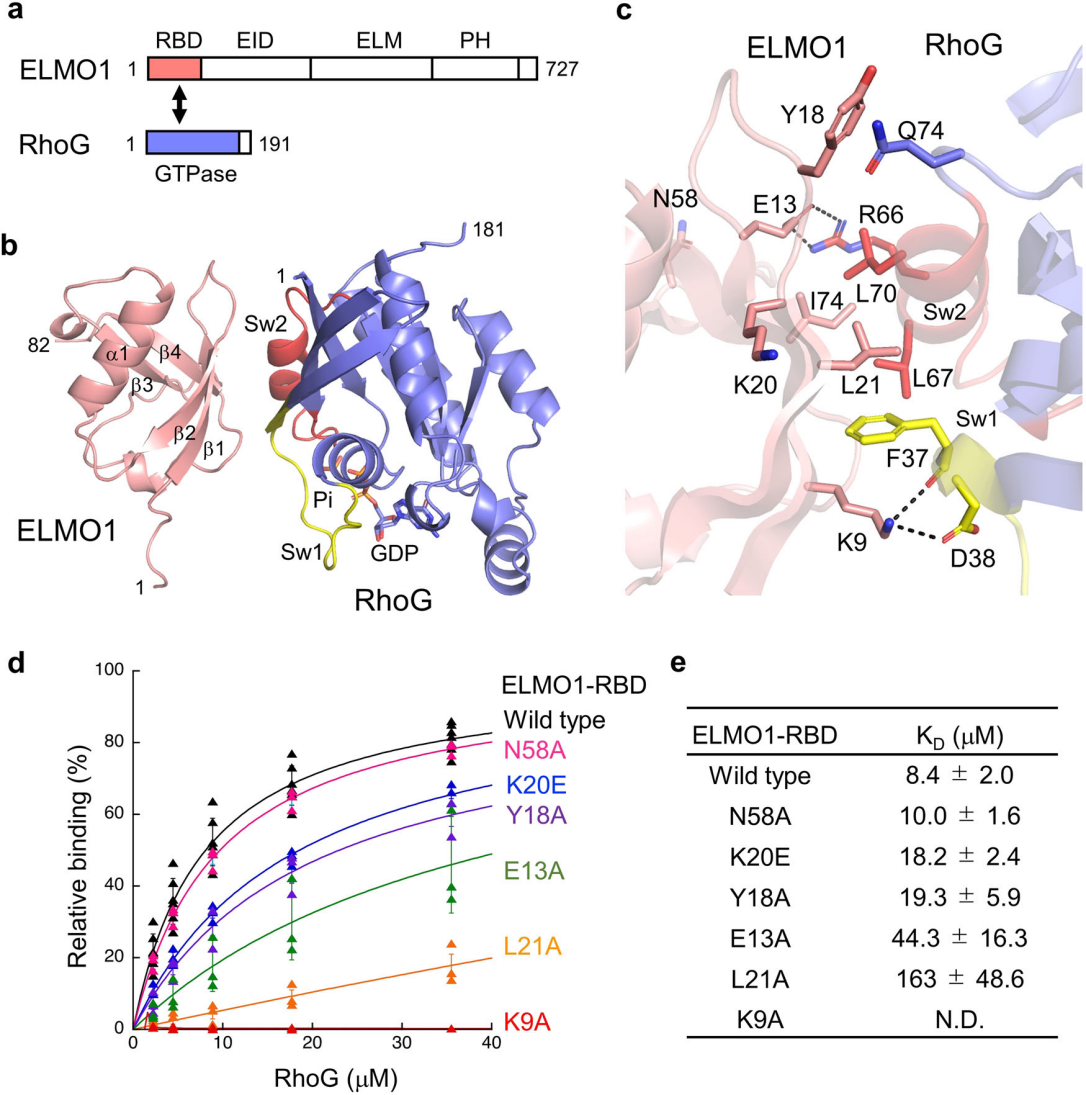
Structure of ELMO1-RBD and its binding to RhoG. **a** Domain organizations of ELMO1 and RhoG. RBD Ras-binding domain, EID ELMO-inhibitory domain, ELM ELMO domain, PH Pleckstrin homology domain. Regions not included in the construct are shown as white boxes. **b** Overviews of the crystal structure of the ELMO1-RBD and RhoG complex. ELMO1-RBD is shown in salmon and RhoG in blue except for switch 1 (Sw1, yellow) and switch 2 (Sw2, red), and bound GDP and Pi are shown as sticks. **c** Interface between ELMO1-RBD and RhoG. Key interacting residues are indicated by stick models. **d** SPR binding analysis between ELMO1-RBD (wild-type and mutants) and RhoG. **e** K_D_ values of ELMO1-RBD for RhoG estimated from SPR binding assay. Data are means ± s.d. (*n* = 3 independent experiments).

A majority of actin-interfering agents discovered are small molecules, which exhibit anti-mitotic and anti-metastatic effects in tumor cells^18,19^. A number of targets in the actin assembly signaling network were exploited by these small molecules while their modes of action can be categorized into the inhibition or enhancement of the polymerization or depolymerization of actin filaments and therefore disrupting the monomer-polymer equilibrium that is required for de novo nucleation or remodeling of existing actin filaments^18^. Alternative to the small molecule class, in a growing trend of antibody-drug development^20^, there were several early examples of novel antibodies that demonstrated anti-tumor effect through their potential actin-interfering actions by targeting actin-binding protein^21^, actin-capping protein^22^ and Ras-effector interaction^23^. In this study, we have determined the structure of ELMO1-RBD alone and in complex with RhoG, and verified its targetability through computational nanobody design. Our rationale of targeting ELMO-RBD by nanobody is, firstly, ELMO1-RBD (1-82), which is a single beta-grasp fold (9 kDa), is essential and sufficient for RhoG binding^6^ (Fig. 1a) and therefore offered a pinpoint feasibility for drugging upstream Rho/Rac1 signaling mediated by ELMO1/DOCK180. Secondly, because there are no obvious binding pockets for small molecule design on the surface of ELMO1-RBD from our observation, it is a particularly suitable use case of antibody design. Thirdly, the ELMO-EID domain flanking C-terminal of ELMO1-RBD forms a curved, bulky scaffold with its armadillo repeats^12,24^, and therefore is prone to major steric hindrance if ELMO1-RBD is targeted by full-length antibody or antibody fragments, such as the Fab or scFv. Nanobody, on the other hand, is a single-domain VHH (~15 kDa). Due to its small size, nanobody allows a higher degree of freedom for epitope and pose selection during computational design.

We applied a dock-and-design approach that emphasized the selection of initial poses demonstrated potential nativeness in terms of resemblance to known nanobody poses and a funnel-shaped binding energy landscape, which led to a detectable binder from the first-round design. With an additional step of computational affinity maturation, multiple nanobody designs that bound ELMO1-RBD in single-digit micromolar K_D_ have been identified. To the best of our knowledge, these are the first reported antibody or nanobody against ELMO1-RBD. This class of nanobody has demonstrated the feasibility of targeting ELMO1-RBD and can be further explored to develop antibody drugs targeting actin assembly through Rac1 activation by ELMO1/DOCK180.

## Results and discussion

### Structure determination of ELMO1-RBD alone and in complex with RhoG

The detailed structure of ELMO1-RBD has not been reported. In this study, we first determined the solution structure of human ELMO1-RBD by NMR experiments. A total of 1589 NOE distance restraints obtained from 3D ^15^N-edited and ^13^C-edited [^1^H, ^1^H]-NOESY spectra were assigned and used in the structure calculation, along with 40 dihedral angle restraints (Table 1). The 40 structures obtained from the CYANA calculation were further refined with the AMBER14 program to yield the final 20 energy-refined conformers (Supplementary Fig. 1). The solution structure showed that residues 7-80 of ELMO1 create the core RBD domain consisting of a four-stranded β-sheet, two 3_10_-helices and one α-helix (β1: Ile7-Glu13, β2: Lys20-Asp25, α1: Leu30-Gly40, 3_10__1: His46-Tyr48, β3: Phe49-His53, 3_10__2: Arg66-Glu68, β4: Thr73-Thr79, respectively). The topology of ELMO1-RBD was a ubiquitin-like fold similar to that of ELMO2-RBD^17^. The electrostatic surface potentials of ELMO1-RBD showed no specific charge distribution (Supplementary Fig. 1), which makes clear contrast to the RBDs of Ras effectors having a positively charged surface for binding to Ras.

**Table 1:** NMR and refinement statistics for ELMO1-RBD.

	ELMO1-RBD
**NMR distance and dihedral constraints**	
Distance constraints	
Total NOE	1589
Intra-residue	483
Inter-residue	
Sequential (|*i*–*j* | = 1)	400
Medium-range (|*i*–*j* | < 4)	205
Long-range (|*i*–*j* | > 5)	501
Hydrogen bonds	48
Total dihedral angle restraints	
χ	40
**Structure statistics**	
Average pairwise r.m.s. deviation**(Å)	
Heavy	1.00
Backbone	0.32

**Pairwise r.m.s. deviation was calculated among 20 refined structures.

Next, we determined the crystal structure of ELMO1-RBD in complex with RhoG at 1.6 Å resolution (Table 2). The asymmetric unit contained four virtually identical complexes of ELMO1-RBD and active form of RhoG. ELMO1-RBD interacted with RhoG via β1, β2, and β4, whereas Raf-RBD, a conventional Ras effector, uses β2 and α1 for binding to Ras (Fig. 1b and Supplementary Fig. 2). The interface buried Leu21 (β2) of ELMO1 in a cleft formed by RhoG switch 1 and 2 regions (Fig. 1c). Nearby, multiple hydrophobic interactions were observed, as well as two electrostatic interactions between Lys9 (β1) of ELMO1 and RhoG switch 1 region and between Glu13 (β2) of ELMO1 and RhoG switch 2 region. To validate the observed interactions, we tested several mutants of ELMO1-RBD by surface plasmon resonance (SPR) binding assays (Fig. 1d, e). Substitution of Lys9, Glu13 and buried Leu21 of ELMO1-RBD with alanine (K9A, E13A, L21A) dramatically reduced its binding affinity to RhoG in the active GTPγS-bound form. Mutations of several residues at the periphery of the interface (Y18A and K20E) also slightly reduced its binding affinity to RhoG, but a mutation at a distal position (N58A) had no effect. These results are consistent with the recently reported mouse ELMO2-RBD and RhoG complex (PDB: 6UKA)^12^. Furthermore, two ELMO1-specific residues, Tyr18 and Pro19, were found to interact with Gln74 and Pro73 of RhoG, respectively. In addition, compared to the ELMO2-RhoG complex, Trp56 of RhoG was in closer contact with Leu21 of ELMO1. Sequence alignment of the Rho family GTPases indicated that all ELMO1-binding residues of RhoG are conserved in Rac1-Rac3 (Supplementary Fig. 2), suggesting the possibility of ELMO1 binding. Indeed, the SPR binding assay confirmed that Rac1 binds to ELMO1-RBD, albeit more weakly than RhoG (Supplementary Fig. 3). The weak binding of ELMO1 to Rac1 is probably due to the substitution of Thr69 in RhoG with Pro69 in Rac1, which breaks α2 helix in switch 2 (Supplementary Fig. 2). Furthermore, Cdc42, in which Trp56 in RhoG/Rac1 is replaced by Phe56, showed even weaker binding to ELMO1-RBD (Supplementary Fig. 3). Thus, the present crystal structure clearly provides structural evidence that ELMO1 binds preferentially to RhoG.

**Table 2:** Crystallographic statistics of the ELMO1-RBD and RhoG complex.

	ELMO1-RBD•RhoG complex
**Data collection**	
Space group	*P*2_1_
Cell dimensions	
* a*, *b*, *c* (Å)	74.7, 63.8, 115.3
α, β, γ (°)	90, 100.5, 90
Resolution (Å)	42.4–1.60 (1.69–1.60) *
* R*_meas_	0.048 (0.541)
* I*/σ*I*	16.8 (2.4)
Completeness (%)	98.6 (98.1)
Redundancy	3.7 (3.8)
**Refinement**	
Resolution (Å)	42.4–1.60 (1.62–1.60)
No. reflections	139937
* R*_work_/*R*_free_	0.198/0.236
No. atoms	
Protein	8264
Ligand/ion	136
Water	1346
*B*-factors	
Protein	34.6
Ligand/ion	20.6
Water	41.5
R.m.s. deviations	
Bond lengths (Å)	0.007
Bond angles (°)	0.971

^*^One crystal was used for the structure determination.

^*^Values in parentheses are for highest-resolution shell.

### Identification of Nb01 as a detectable binder from first-round design

**Fig. 2:**
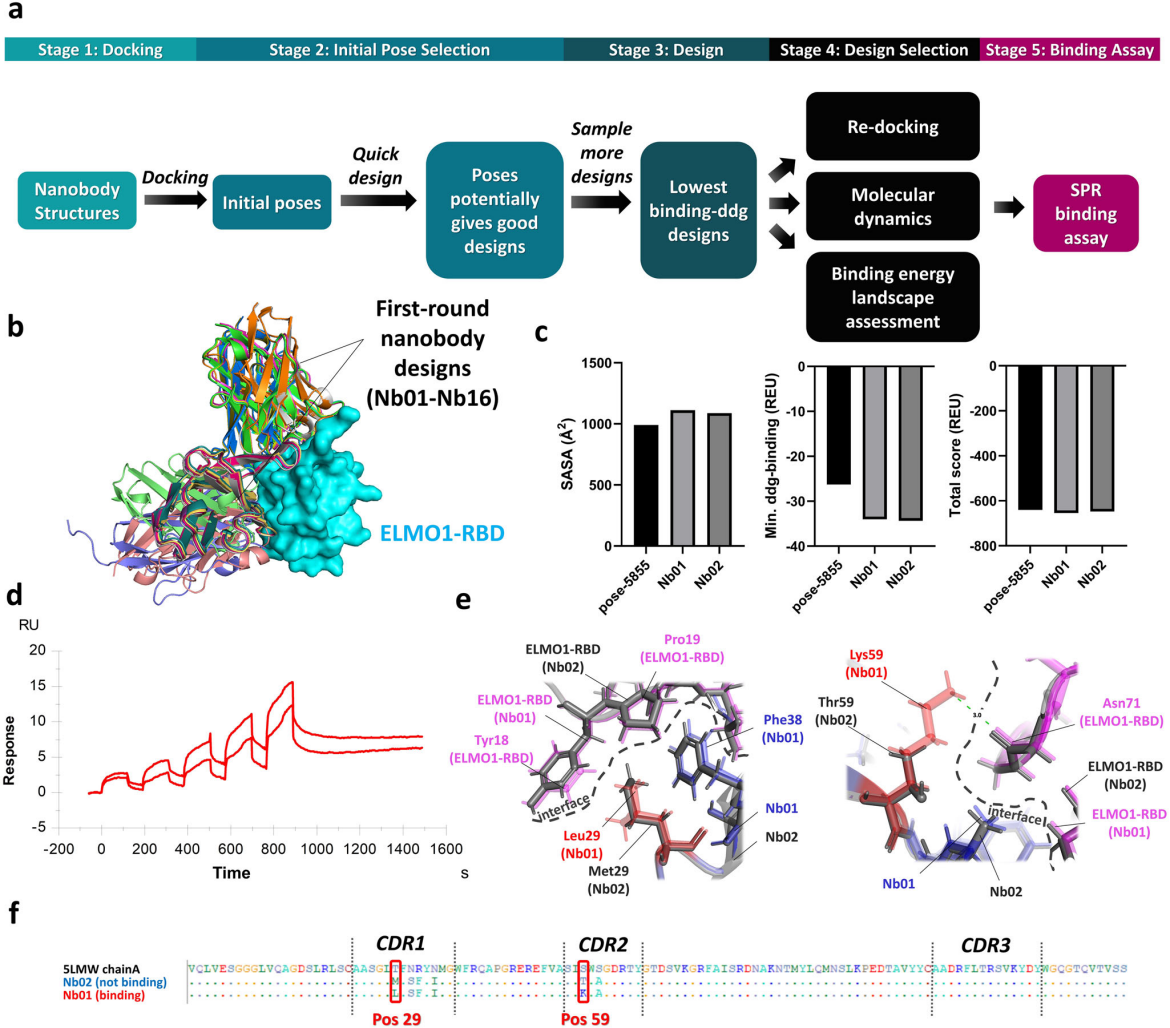
Computational design and experimental verification results from first-round design. **a** Overall design workflow in first-round design. **b** Superposition of first-round nanobody designs revealed the stabilization of two clusters of binding poses on two distinct epitopes on ELMO1-RBD. **c** Rosetta energy scores and buried solvent accessible area comparisons between designs Nb01, Nb02 and pose-5855. **d** SPR single-cycle kinetics of Nb01 binding to ELMO1-RBD. Nb01 was tested at concentrations of 0.5, 1, 2, 4, and 8 μM from left to right (*n* = 2 independent experiments). **e** Comparison of side chain interactions at position 29 and 59 between Nb01 and Nb02 at their interfaces with ELMO1-RBD. **f** Comparison of design mutations between Nb01 and Nb02 with reference to the sequence of parent nanobody PDB 5 LMW. Dots represent the same residue type as the parent nanobody at the aligned positions.

Our first-round computational nanobody design targeting ELMO1-RBD, which was a dock-and-design workflow that emphasized initial pose selection (Fig. 2a), has yielded 16 design candidates, sequentially named Nb01 to Nb16 (Fig. 2b) that were originated from five initial poses selected from the workflow. Although we did not specify distance constraints to any epitope residues on ELMO1-RBD during docking, two clusters of binding pose of the design candidates emerged, which revealed the potential targetability of the two ELMO1-RBD epitopes using our design workflow. Apart from the binding poses, we verified the design interfaces at the atomic level by confirming the de novo design of strong interactions, which were all located on the three CDR loops (CDR1, CDR2 and CDR3) (Supplementary Fig. 4) and therefore the favorable enlargement of the buried solvent accessible area (SASA) together with an enhanced predicted ddg-binding score while without affecting the overall stability of the nanobody (Fig. 2c).

The 16 design candidates were tested by SPR binding assay, which has led to the identification of Nb01, a hit showing weak but detectable binding to ELMO1-RBD (Fig. 2d). We traced back the design path of this hit. Nb01 was designed from the parent nanobody chain A of PDB 5LMW, which has a native antigen unrelated to ELMO1. In contrast to the common practice of selecting initial poses that ranked top (e.g. top 10^th^–100^th^) by the complementarity score from PatchDock for design^25–27^, pose-5855, the initial pose of the parent nanobody that gave rise to Nb01, ranked 355^th^ in a total number of 586 initial poses sampled by PatchDock. The successful design of a mid-to-low-ranked initial pose into a hit demonstrated the ability of our initial pose selection strategy in enriching native-like poses for design.

To understand the structure-activity relationship of the binding of Nb01 to ELMO1-RBD, we compared the designed side-chain interactions of Nb01 versus Nb02, a sequence variant forked from the design path of pose-5855 but failed to bind ELMO1-RBD (Fig. 2e). Nb01 and Nb02 only differed in their sequences by two design mutations at position 29 (T29L_Nb01_ and T29M_Nb02_) and position 59 (S59K_Nb01_ and S59T_Nb02_) (Fig. 2f), which were located at CDR1 and CDR2 respectively. From the design interfaces, compared with Met29_Nb02_, hydrophobic interactions with hydrophobic rings of Pro19_ELMO1-RBD_, Tyr18_ELMO1-RBD_ and Phe38_Nb01_ were potentially favorable with the hydrophobic side chain of Leu29_Nb01_. Besides, Thr59_Nb02_ disrupted a predicted 3 Å hydrogen bond designed between Lys59_Nb01_ with Asn71_ELMO-RBD_. These observations provided us valuable structure-activity information that guided the subsequent affinity maturation of Nb01.

### Improved ELMO1-RBD binding by Nb01 sequence variants through computational affinity maturation

**Fig. 3:**
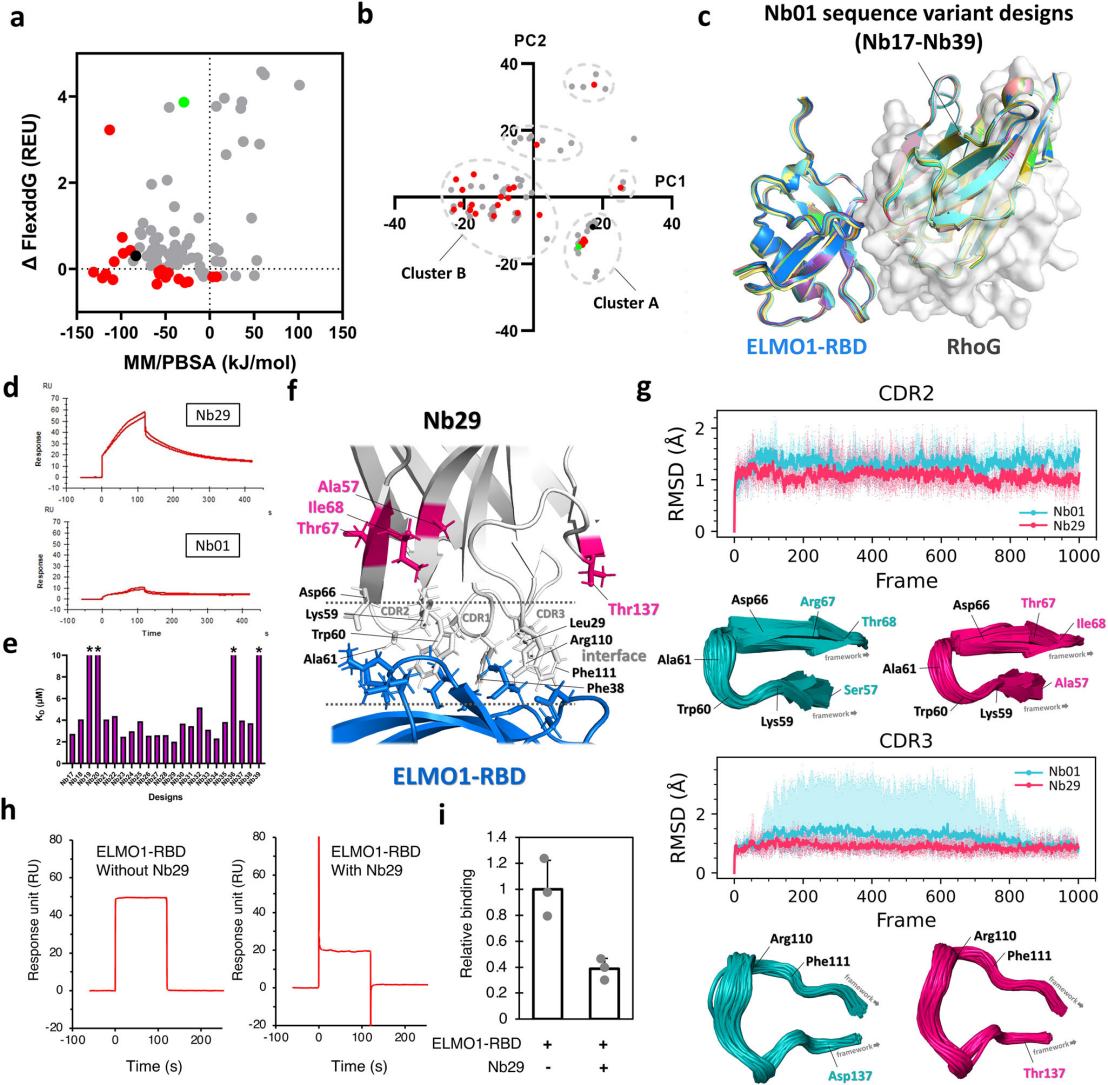
Computational design and experimental verification results from affinity maturation design of Nb01. **a** Comparison of ΔFlexddG and MM/PBSA between Nb01 sequence variants selected (red) and unselected (grey) by binding affinity estimation with reference to Nb01 (green) and Nb02 (black). **b** Relative positions in local sequence space among Nb01 sequence variants selected (red) and unselected (grey) as the final candidates from affinity maturation design with reference to Nb01 (green) and Nb02 (black). **c** Spatial overlap between the final candidates from affinity maturation design with RhoG according to the modeled nanobody-ELMO1-RBD complexes. **d** Comparison of SPR sensorgrams of Nb29 and Nb01 at 2 μM concentration (*n* = 2 independent experiments). **e** Measured K_D_ values between the final candidates from affinity maturation design to ELMO1-RBD from SPR binding assay. Asterisks represents K_D_ > 1e–5 M. **f** The newly designed mutations after affinity maturation (pink) in Nb29 were distant from the interface with ELMO1-RBD. **g** Comparison of loop flexibility of CDR2 (top) and CDR3 (bottom) between Nb29_apo_ (pink) and Nb01_apo_ (cyan) from molecular dynamics simulation. Locations of Cα atoms of the interface residues (black) and CDR-framework designed residues (color of the respective design) were annotated on the CDR loops. For the line plots, the solid lines and the shaded regions represent the mean and the range of RMSD of the CDR loops in snapshots extracted from independent production runs (*n* = 5), respectively. **h** Binding of Nb29 to ELMO1 suppressed RhoG/ELMO1-RBD interaction. Competitive binding assays comparing SPR sensorgrams of ELMO1-RBD without and with an excess of Nb29 against immobilized active RhoG. The concentrations of ELMO1-RBD and Nb29 were 5 μM and 500 μM, respectively. **i** Normalized binding of ELMO1-RBD to active RhoG. Data are means ± s.d. (*n* = 3 independent experiments).

With Nb01 as the hit and the structure-activity relationship, we performed computational affinity maturation by designing additional Nb01 sequence variants to improve its binding affinity to ELMO1-RBD. Because we focused on the exploration of a very broad CDR sequence and conformational space in our first-round design (Supplementary Fig. 5), we were aware of the inadequate exploration of sequence variants surrounding the local sequence space of Nb01. Therefore, we took a balanced exploration-exploitation design approach, such as to design upon the Nb01 decoy (exploitation) and also the pose-5855 decoy (exploration), which were performed in parallel through our design scripts covering 16 parameter variations described in the Materials and Methods section. We successfully sampled new Nb01 sequence variants that showed better predicted binding abilities in terms of MM/PBSA and FlexddG scores (Fig. 3a) and all local sequence clusters were covered (Fig. 3b), fulfilling our design objective to balance exploration-exploitation. Because MM/PBSA and FlexddG represented two different methods of binding affinity prediction, a few designs were predicted with good affinity by one method and poor affinity by the other as shown in Fig. 3a. A greater number of backrub steps (e.g. >10,000 steps) in the FlexddG simulation may improve the consistency of results between the two methods. However, a 10,000-step backrub is adequate for our purpose as two alternative methods were used for binding affinity prediction and a majority of designs showed a trend of consistency of the predicted affinity by the two methods. Besides, we confirmed the almost identical (RMSD_Cα_ < 0.3 Å) binding poses between Nb01 and each of the 23 Nb01 sequence variants (sequentially named Nb17 to Nb39), which were all predicted to form substantial spatial overlap with RhoG in the ELMO1-RBD/RhoG complex structure (Fig. 3c) and therefore could potentially compete with RhoG for ELMO1-RBD binding.

The SPR binding assay has confirmed that 17 out of 23 Nb01 sequence variants showed enhanced binding affinity to ELMO1-RBD compared with Nb01 (Fig. 3d, e). Multiple Nb01 sequence variants bound ELMO1-RBD at single-digit μM K_D_ and the best K_D_ was 2.0 μM by the sequence variant Nb29. We again traced back the design and selection processes to evaluate how the newly designed mutations have contributed to the enhanced binding affinity of the Nb01 sequence variants to ELMO1-RBD. The top 10 sequence variants that showed the strongest binding to ELMO1-RBD belonged to sequence cluster B while Nb01 and Nb02 sampled from first-round design belonged to sequence cluster A (Fig. 3b), demonstrating the successful exploration of an alternative local sequence space that were enriched with sequence variants of improved binding to ELMO1-RBD with our MM/PBSA and FlexddG scoring. Besides, we re-confirmed, within the sequence space explored, the importance of Leu29 on CDR1 and Lys59 on CDR2 of our designs because all designs within the top 50% binding affinity to ELMO1-RBD binding harbored these designed residues.

Surprisingly, according to the designed interface, the newly designed mutations on the best binder Nb29 relative to Nb01 (S57A, R67T and T68I on CDR2 and D137T on CDR3) did not form direct contacts to ELMO1-RBD but were located at the CDR-framework junctions of Nb29 (Fig. 3f), which triggered our curiosity to investigate how the designs of the CDR-framework junctions have enhanced ELMO1-RBD binding of Nb29 compared to Nb01. We tested our hypothesis of the potential alteration in flexibility of CDR2 and CDR3 that contributed to the enhanced binding affinity of Nb29 by molecular dynamics simulation. We identified the subtle but observable stabilizations at the bound conformation of both CDR2 and CDR3 of Nb29_apo_ compared with Nb01_apo_ (Fig. 3g and Supplementary Fig. 6). In one of the production runs, the CDR3 backbone of Nb01_apo_ destabilized to an RMSD > 2 Å between 2^nd^ to 6^th^ ns while that of Nb29_apo_ remained relatively stable. The more stable CDR2 and CDR3 potentially contributed to the decreased entropy penalty due to a reduced loss of loop flexibility upon ELMO1-RBD binding in Nb29. Additionally, it was previously shown the requirement of high energy and contact density interactions in native nanobody-antigen interfaces^28,29^ and it is plausible a more stabilized CDR2 and CDR3 contributed to a more stable core of side chains packing at the interface of Nb29 with ELMO1-RBD.

### Improved nanobody suppresses RhoG/ELMO1 interaction

To determine whether the improved nanobody could inhibit the RhoG/ELMO1 interaction, competitive binding assays were performed using purified proteins. The best binder Nb29 was added in excess in the SPR binding assays of ELMO1-RBD to active RhoG and its effect on binding was examined (Fig. 3h, i). Whereas ELMO1-RDB without Nb29 specifically bound to the GTPγS-bound active RhoG, the addition of Nb29 substantially reduced the binding, indicating that Nb29 inhibited the interaction between ELMO1-RBD and RhoG.

### Dock-and-design approach emphasizing initial pose selection

The dock-and-design approach is an experimentally verified protein-protein interaction design strategy^25–27,30–33^, thus was adopted as the overall strategy of our computational nanobody design workflow. However, because computational antibody design is an antibody discovery strategy still suffering from a low success rate^34,35^, we explored two strategies of initial pose selection in an attempt to enrich initial poses that were more promising to produce a hit. We reasoned that the initial pose selection is of great importance in the dock-and-design approach because, without an initial pose of modeled protein-protein complex that can be feasibly designed into a native interface, the subsequent design effort is futile. Moreover, protein-protein docking often generates a considerable number of alternative solutions of docked poses, therefore an efficient initial pose selection method is critical to confine the number of poses manageable for design simulation and binding assay.

**Fig. 4:**
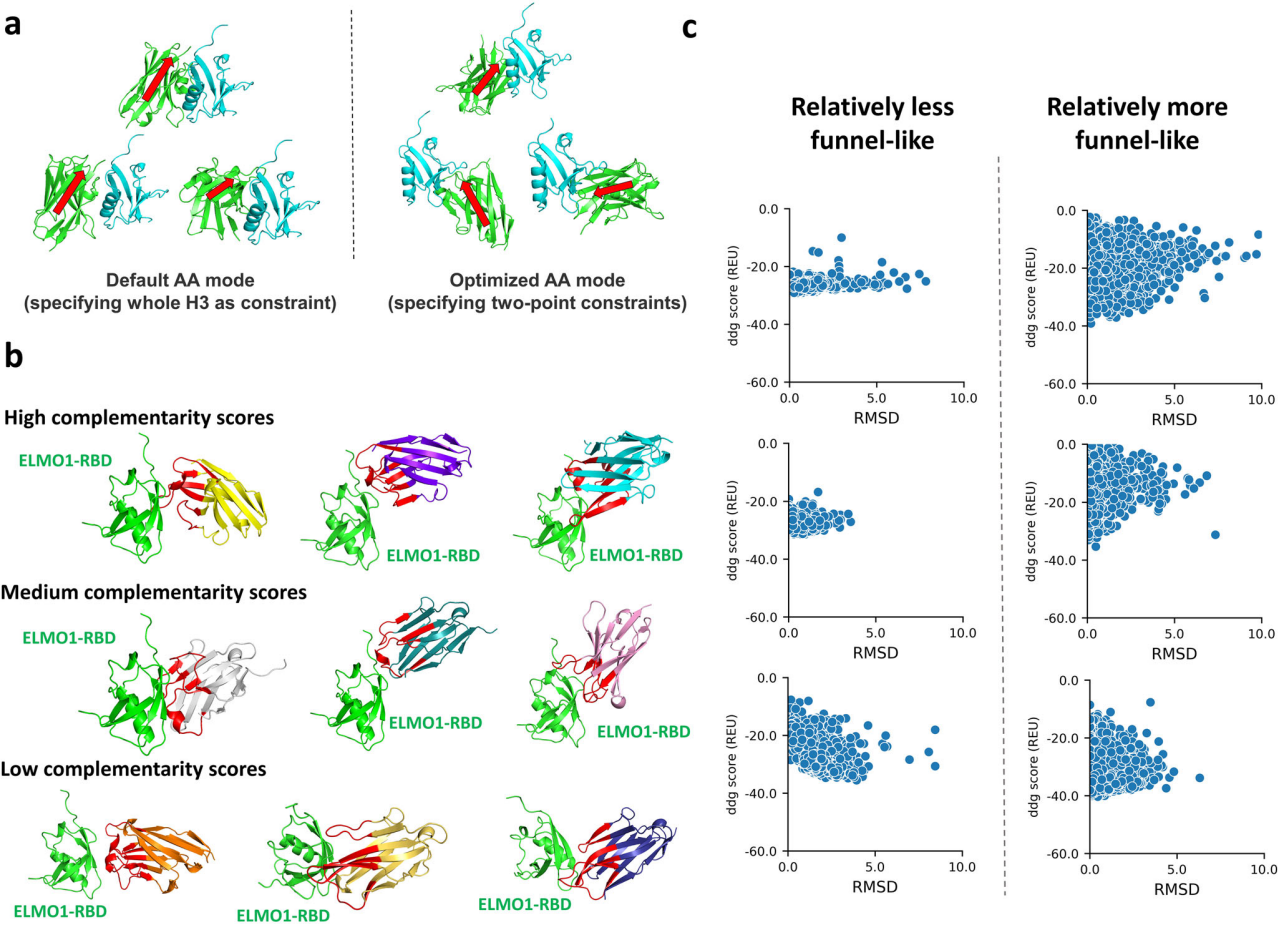
Initial pose selection strategy in first-round design. **a** Comparison of initial poses of nanobody (green) binding to ELMO1-RBD (cyan) generated by the default antibody-antigen (AA) mode and the optimized AA mode of PatchDock. Arrows (red) are approximately in parallel to the beta-strands of framework and are pointing towards the CDR loops. **b** Illustration of initial poses generated from the optimized AA mode in PatchDock with respect to their range of PatchDock complementarity scores among all compatible initial poses. **c** Comparison of the relatively less funnel-like and relatively more funnel-like binding energy landscape sampled by RosettaDock.

Firstly, we successfully automated the generation of initial nanobody poses that showed high resemblance to known nanobody-antigen complexes by imposing and optimizing the CDR distance constraints in the antibody-antigen mode of PatchDock (Fig. 4a). We observed that considerable proportions of nanobodies bind their native antigens with framework beta-strands pointing at a high angle and sometimes close to perpendicular against the epitope surface. This is consistent with the previously described major binding mode of nanobody to antigens such that all three CDRs should form contact with the epitope^29,36^, which often render the observed high-angle orientation of nanobody. This has led us to an optimized antibody mode of PatchDock that offered a > 10-fold enriched set of initial poses that a majority of them reconstituted the native-like CDR-epitope contact (Supplementary Fig. 4). Regardless of the range of complementary score of PatchDock, the majority of initial poses were, to our best judgment, visually indistinguishable from native poses of known nanobody-antigen complexes (Fig. 4b).

Secondly, we applied a binding energy landscape selection strategy during both initial pose selection and design selection to identify designs that showed signs of nativeness by favoring the funnel-shaped binding energy landscape (Fig. 4c). The existence of a funnel-shaped binding energy landscape in protein-protein and antibody-antigen interfaces was well-documented^37–42^ and therefore was generally employed to distinguish native from non-native protein-protein docking solutions^43–47^. Theoretically, the use of binding energy landscape for initial pose selection in the design context is more challenging because the initial poses are to-be designed (i.e. before sequence optimization) and therefore, even for poses showing good complementarity to the epitope, should give relatively shallow funnel-shaped landscapes due to a less-than-optimum side-chain packing, which normally corresponds to the steep energy drop within <2 Å RMSD_Cα_ from the native pose^48^. Moreover, among these native-like binding energy conformations, they are contaminated by a greater proportion of non-native binding energy conformations because, in contrast to ordinary protein-protein docking where binding is often verified a priori, the existence of a hit among the designs is unknown. Yet, several successful dock-and-design examples have demonstrated the high consistency between the initial pose and the experimentally determined structure of their ultimate designs^25,31–33^. It means that native-like poses before sequence optimization can be indeed sampled by the initial docking, which inspired our attempt to isolate native-like from non-native-like initial poses using a binding energy landscape selection approach.

## Conclusion

We have determined the structures of ELMO1-RBD alone by NMR spectroscopy and in complex with RhoG by X-ray crystallography, which have paved the way for the subsequent computational studies. Through computational nanobody design, we successfully generated a class of nanobodies that bind to ELMO1-RBD, the Ras-binding domain involved in actin assembly activation through ELMO1/DOCK180, validating the targetability of this functionally important domain for the further development of therapeutics modulating actin dynamics. We suggested alternative approaches in initial nanobody pose selection for dock-and-design and described in detail how to select native-like nanobody poses and the funnel-shaped binding energy landscapes, serving as an added example of methodological exploration in the computational design approach for antibody discovery. Further optimization of the Nb sequence will be needed in the future to assess ELMO1 inhibition by nanobody design in cells.

## Materials and methods

### Plasmids

DNA fragments of human ELMO1-RBD (residues 1–82, 1–98, and 1–113) were cloned into the expression vector pCR2.1 (Invitrogen). A histidine tag and a TEV cleavage site were attached to the N-terminus of ELMO1-RBD. The gene encoding human RhoG (residues 1–184) was cloned into pDEST10.1 (Invitrogen) with an N-terminal poly-histidine tag and a TEV cleavage site. The genes encoding human Rac1 (residues 1–177) and Cdc42 (residues 1–178) were cloned into pCR2.1 with an N-terminal histidine tag and a TEV cleavage site^49,50^. pRK793, an expression plasmid for TEV protease^51^, was a gift from David Waugh (National Cancer Institute, Frederick, MD, USA).

### Protein expression and purification

ELMO1-RBD was expressed using the *Escherichia coli* cell-free protein synthesis^52,53^ and purified using a HisTrap column (Cytiva). The His-tag was removed by cleavage with TEV protease, overnight dialysis, and another passage through the HisTrap column. Protein was further purified by ion exchange chromatography on a HiTrap Q column (Cytiva), and finally on a HiLoad 16/600 Superdex 75 column (Cytiva). Protein was concentrated to 10 mg/ml in 20 mM sodium phosphate buffer (pH 7.0) containing 100 mM NaCl. Rac1 and Cdc42 were expressed using the *E. coli* cell-free protein synthesis and purified in the same manner. For NMR spectroscopy, ^13^C/^15^N-labeled protein was expressed using the *E. coli* cell-free reaction and purified similarly as the non-labeled protein. ^13^C/^15^N-labeled ELMO1-RBD were concentrated to 1.0 mM in 20 mM Sodium Phosphate buffer (pH 7.0), containing 100 mM NaCl, 1 mM DL-dithiothreitol-d_10_ (*d*-DTT) and 0.02% NaN_3_ (in 90% H_2_O/10% D_2_O). For purification of RhoG, the baculovirus was created using the Bac-to-Bac Baculovirus Expression System (Invitrogen), and was used to infect *Spodoptera frugiperda* (Sf9) cells. Cells were harvested 48 h after infection, resuspended in 20 mM Tris-HCl (pH 8.0), 500 M NaCl, 10% glycerol, and 20 mM imidazole, and then disrupted by sonication. After centrifugation, the cleared lysate was loaded on a HisTrap column, and then the protein was eluted with a linear gradient of 20–500 mM imidazole in 20 mM Tris-HCl (pH 8.0), 500 M NaCl, and 10% glycerol. The eluate was supplemented with TEV protease, dialyzed overnight at 4 °C, and passed through the HisTrap column to remove His-tag. The protein sample was further purified by size-exclusion chromatography on a HiLoad 16/600 Superdex 75 column in 20 mM HEPES-NaOH (pH 7.5), 150 mM NaCl, 10% glycerol, and 2 mM DTT.

DNA fragments coding designed nanobodies were synthesized using gBlooks gene fragments (Integrated DNA Technologies). They were expressed using a previously described high-throughput method of cell-free protein synthesis^54^. Briefly, the PCR reaction was preformed to attach the sequences for the T7 promoter, a natural poly-histidine tag, and a TEV cleavage site at the N-terminus, and the sequence for the T7 terminator at the C-terminus of the nanobody design sequence. Using the PCR products as the template, the cell-free protein synthesis reaction was carried out at 25 °C for 8 h in the presence of 12 mg/ml Gam protein^55^, 19 mg/ml disulfide isomerase (DsbC), and 100 mM oxidized glutathione (GSSG)^56^. Each nanobody design was purified by Ni Sepharose resin (Cytiva) and used for SPR binding assay.

### NMR spectroscopy

NMR experiments were performed at 298 K on Bruker AVIII- 700 and 900 MHz spectrometers equipped with a 5 mm triple resonance probe (CryoProbe). Assignments of protein resonances were performed by 2D [^1^H, ^15^N]-HSQC, 2D [^1^H, ^13^C]-HSQC, 3D HNCO, 3D HN(CA)CO, 3D HNCACB, 3D CBCA(CO)NH, 3D H(CCCO)NH, 3D (H)CC(CO)NH, 3D ^15^N-edited [^1^H, ^1^H]-NOESY and ^13^C-edited [^1^H, ^1^H]-NOESY spectra. 3D NOESY spectra were recorded with mixing times of 80 ms. Pulse programs of 2D HSQC and 3D NOESY spectra were modified from those existing in-house^57^ (Supplementary Notes 1 and 2). All NMR data were processed using the NMRPpipe or Topspin software (Bruker) and were analyzed with the NMRView and KUJIRA programs.

### NMR structure calculation

The three-dimensional structure of ELMO1-RBD (residues 1–113) was determined by combining the automated NOESY cross-peak assignment and the structure calculations with torsion angle dynamics implemented in the program CYANA 2.1. Dihedral angle restraints for χ angle were obtained from analyzing the pattern of the inter- and intra-NOE intensities of 3D NOESY spectra. The structure calculations started from 200 randomized conformers and used the standard CYANA simulated annealing schedule, with 40,000 torsion angle dynamics steps per conformer. The 40 conformers with the lowest final CYANA target function values were further refined with the AMBER14 program^58^, using an Amber ff14SB force field and a generalized born solvation model, as previously described^59^. The 20 conformers that were most consistent with the experimental restraints were then used for further analyses. PROCHECK-NMR and MOLMOL programs were used to validate and to visualize the final structures, respectively. Ramachandran statistics are 89.3%, 9.9%, 0.3%, and 0.5% for residues in most favored, allowed, generously allowed, and disallowed regions, respectively.

### Crystallization and structure determination

Equimolar amounts of ELMO1-RBD (residues 1–82) and RhoG were mixed at 16 mg/ml and incubated at 4 °C for 4 h prior to crystallization. 1 μl of protein solution were mixed with 1 μl of reservoir solution. The best diffraction crystals of the ELMO1-RBD and RhoG complex were obtained in 0.91 M potassium phosphate dibasic and 0.49 M sodium phosphate monobasic monohydrate at 20 °C by the hanging-drop vapor diffusion method. Crystals were flash cooled in liquid nitrogen in the reservoir solution. X-ray data were collected at 100 K at a wavelength of 1.0 Å at SPring-8 beamline BL26B2 (Harima, Japan). Data were processed using the XDS program^60^. The structure was determined by the molecular replacement method with the Phaser program^61^ using the solution structure of ELMO1-RBD (this study) and the crystal structure of H-Ras-GppNHp bound to the Raf RBD (PDBID: 4G0N) as the search models. The model was built and refined iteratively using Coot^62^ and Phenix^63^ programs, and its quality was evaluated using PROCHECK software^64^. Molecular graphics were generated using the PyMOL program^65^. Ramachandran statistics are 92.7%, 7.1%, 0.2%, and 0.0% for residues in most favored, allowed, generously allowed, and disallowed regions, respectively.

### SPR binding assay

SPR experiments were performed with a Biacore T200 instrument (Cytiva). To validate the crystal structure of the ELMO1-RBD and RhoG complex, GTPγS-bound RhoG was prepared by incubating 60 μM RhoG with 5 mM EDTA and 1 mM GTPγS at 4 °C for 3 days, stopping the reaction by adding 10 mM MgCl_2_, and removing excess guanine nucleotides by ultrafiltration. An N-terminal glutathione S-transferase (GST)-tagged ELMO1-RBD (residues 1–113, wild-type or mutants) or GST alone as a negative control was immobilized on to a CM5 Sensor Chip using a GST Capture Kit (Cytiva). Five different concentrations (2.2–35.5 μM) of GTPγS-bound RhoG were injected, and the response was measured in 10 mM HEPES (pH 7.5), 150 mM NaCl, 1 mM MgCl_2_, and 0.005% surfactant P-20. Similarly, responses between ELMO1-RBD and other Rho GTPases, Rac1 and Cdc42, were also measured. Data were analyzed by steady-state affinity analysis using the manufacturer’s software.

For nanobody design selection, ELMO1-RBD (residues 1–98) was immobilized on a CM5 Sensor Chip using an Amine Coupling Kit (Cytiva). The buffer contained 10 mM HEPES (pH 7.5), 150 mM NaCl, and 0.005% surfactant P-20. Five different concentrations (0.5–8 μM) of each nanobody design were injected continuously and the response was measured. Data were processed by single-cycle kinetic analysis using the manufacturer’s software.

In the competitive binding assays, active (GTPγS-bound) RhoG was immobilized in the ligand flow cell of the CM5 Sensor Chip using an Amine Coupling Kit. Inactive (GDP-bound) RhoG was similarly immobilized in the reference flow cell. ELMO1-RBD (5 μM) or blank buffer was injected alone or with excess of Nb29 (500 μM) and the response was measured in 10 mM HEPES (pH 7.5), 150 mM NaCl, 1 mM MgCl_2_, and 0.005% surfactant P-20.

### Initial pose generation

At first, 490 PDB IDs of known nanobody structures were obtained from the SAbDab database^66^ in Feb., 2019. To ensure a more thorough search of nanobody structures, PDB 5DA4 chain B, which showed the highest sequence identity to the most dissimilar nanobody sequences, was selected as a representative nanobody and used to search against the RCSB Protein Data Bank (March, 2019) by a cut-off of >70% sequence coverage and >30% sequence identity. Sequence similarity instead of structural similarity was used for searching because many unrelated proteins harbor a immunoglobulin-like beta-sandwich superfold. During the search, only nanobody chains that were (1) sequence non-redundant, (2) from X-ray crystal structures better than 2.5 Å resolution, (3) with no missing CDR loops, (4) only from camelid species (Camelidae, Camelus Dromedarius, Lama, Lama Glama and Vicugna Pacos) and (5) with molecular weight <20 kDa, were retained, giving a total of 164 nanobody chains. All nanobody chains were renumbered using the AHo numbering scheme^67^ by PyIgClassify^68^ and relaxed and repacked by Rosetta^69^. ELMO1-RBD structure was isolated from the crystal structure of ELMO1-RBD/RhoG complex. The collected nanobody chains were then docked against any arbitrary surfaces on the ELMO1-RBD structure with an optimized antibody-antigen mode of PatchDock^70^, which specified a two-point distance constraint (residue number 38 and 60 on CDR1 and CDR2 respectively). This two-point distance constraint was derived from our in cerebro nanobody pose learning as we sought to improve the resemblance of docked nanobody-RBD complexes to known nanobody-antigen structures. Distance constraints on CDR3 were not included due to its high conformational diversity and, when constrained, caused unsatisfactory contacts between the framework residues and ELMO1-RBD in a considerable number of initial poses. At first, a total of 39,781 initial poses that modeled the binding of the nanobody chains to ELMO1-RBD were generated. Clashscore, an atom contact measure from PHENIX^71^, was then used to trim the initial poses down to 9799 compatible initial poses which have the nanobody forming substantial spatial overlap with RhoG (clashscore ≥30) and minimal steric hindrance with the armadillo repeats (80–520) of ELMO1 (clashscore <10).

### Initial pose selection

To further confine the number of initial poses, the funnel-shaped energy landscape selection strategy was applied here that assessed a rough binding energy landscape represented by the RMSD-ddg binding score scatter plot from a quick design of 100 structures from each compatible initial pose using a design script described in the “First-Round Design and Design Selection” section below. Binding energy landscapes that showed less funnel-like characteristic were discarded if they failed to satisfy any of the following requirements of the binding energy landscape: (1) successfully generated more than 50 designs; (2) standard deviations of designed nanobody backbones RMSD, which was referenced to the compatible initial pose, were within the highest 50% among all compatible poses; (3) standard deviation of ddg-binding scores of the designed interfaces were within the highest 50% among all compatible poses and (4) backbone RMSD of the minimum ddg-binding score design was less than the average RMSD of all designs sampled from a compatible initial pose. The rationale of applying these filters was to, firstly, disfavor binding energy landscapes that were either or both too narrow or too shallow, which violate the commonly observed size^72^ of the characteristic funnel-shaped native binding energy landscapes. Secondly, binding energy landscapes with the minimum ddg-binding score design being too far from the initial pose were also disfavored because it implied the disagreement between the initial pose generated by PatchDock and the pose optimization by RosettaDock during design simulation. After this binding energy landscape selection, 140 initial poses remained. We scaled up the design upon these 140 initial poses by sampling 5000 designs from each pose and repeating the selection of funnel-shaped binding energy landscape but, instead of selection by the four requirements of binding energy landscape mentioned above, it was performed through direct visual assessment to eliminate less funnel-like binding energy landscapes to yield a final total of 13 selected initial poses.

### First-round design and design selection

The first-round design was performed upon the 13 selected initial PatchDock poses. An in-house nanobody design RosettaScripts^73^, which consisted of a coarse-grain docking of the initial pose by RosettaDock^74^ and a subsequent fixed backbone sequence design step on the CDR residues within 10 Å from the docked nanobody-ELMO1-RBD interface was used as the default nanobody sequence design method throughout the study. The rationale of performing RosettaDock coarse-grain docking after PatchDock was to further search for compatible poses locally <4 Å, which was the RMSD used by PatchDock to cluster its output docking poses. Then, during sequence design, mutations in beta-strands were avoided to prevent potential stability reduction of the CDR loops due to the loss of the secondary structure. Additionally, the design script automatically discarded designs with buried SASA of the interface less than 800 Å^2^, which is the default SASA cutoff in RosettaScripts for protein interface design and the mean paratope buried SASA of known nanobody-antigen complexes^75^. From this RosettaScripts, a total of 260 designs, which were the lowest 20 ddg-binding score designs out of the 5000 designs sampled from each of the 13 selected initial poses, were passed to the first-round design final selection scheme (Supplementary Table 1). The first-round final design selection scheme consisted of selection criteria sets 1 to 12, which included binding affinity estimation by MM/PBSA and visual assessment of the binding energy landscape to optimize the diversity of the final design candidates. Top designs from each criteria set were picked in a cycled order from set 1 to set 12 until 16 sequence non-redundant designs were selected as the first-round design final candidates for SPR binding assay.

### Molecular dynamics simulations and re-docking of designs to ELMO1-RBD

All molecular dynamics simulations used for MM/PBSA binding affinity estimation between nanobody designs and ELMO1-RBD and the flexibility assessment of CDR loops of Nb01_apo_ and Nb29_apo_ were performed using GROMACS version 5.0.4^76^ with AMBER99SB forcefield^77^ and the following settings. Solvation was performed using TIP 3-point as water model in a dodecahedron box, leaving 10 Å from the protein structure to the edge of the box. The choices of forcefield and water model followed previous studies used for evaluating binding energy of protein-protein and antibody-antigen interactions^78–81^. Net charges were balanced with sodium ions and chloride ions as counterions. Energy was minimized with the steepest descent algorithm in 50,000 steps. Time length of production run was 10 ns with 2 fs per integration. 10 ns is a timescale that can sample conformational changes of CDR loops for the evaluation of their binding to epitopes^82–84^. For MM/PBSA binding affinity estimation, 50 snapshots spanning every 200 ps interval of 0th–10th ns were used for the MM/PBSA calculation by one-step mode of g_mmpbsa^78^. For flexibility assessment of CDR loops, superposition was performed on 100 snapshots spanning every 100 ps interval. RMSD of the CDR loops was calculated upon their backbone Cα atoms using MDAnalysis^85^.

Re-docking of the designed nanobody to ELMO1-RBD by RosettaDock was used for binding energy landscape assessment, minimum ddg-binding score and SASA calculations in the first-round final design selection scheme. Nanobody designs were re-docked to ELMO1-RBD with a centroid mode as the initial step and a subsequent full-atom optimization mode by RosettaDock. Rosetta energy score function ref15sfxn was used in all energy minimization steps. A “funnel visual score” that assessed the shape of binding energy landscapes ranging from the least funnel-like (score = 0) to the most funnel-like (score = 10) was used to assess the binding energy landscapes from re-docking. Consistent with the assessment of the shape of funnel-like binding energy landscape during initial pose selection, the scoring disfavored narrow and shallow binding energy landscapes which had the minimum ddg-binding score design too far (typically, RMSD_Cα_ > 2 Å) from the designed pose.

### Affinity maturation design and design selection

Sixteen parametric variations of the first-round design RosettaScripts were generated through all combinations of the following binary design parameters: (1) either forcing the design of Leu29 and Lys59 or not, (2) either designing all CDRs or only H1 and H2, (3) either using parent nanobody (PDB 5LMW) or Nb01 as the starting structure for design and (4) either designing residues 10 or 12 Å from the interface. The reason for using these combinations of design parameters was to increase the design sequence diversity to cover a reasonable size of local sequence space surrounding Nb01. From each variation of the design RosettaScripts, the lowest 100 ddg-binding score sequence variants out of 5000 were retained, which accounted for an initial total of 1600 and converged to a final total of 89 non-redundant Nb01 sequence variants. The non-redundant Nb01 sequence variants were passed to binding affinity estimation by MM/PBSA and FlexddG^86^. FlexddG score was calculated by generating ten structures with a maximum of 1000 minimization iterations and 10,000 backrub trials in a trajectory stride of 2500. The difference of FlexddG scores was then calculated by subtracting the FlexddG score of each non-redundant Nb01 sequence variant from that calculated from Nb01 as reference. Initially, 20 Nb01 sequence variants were selected according to the affinity maturation design final selection scheme (Supplementary Table 2), where the best designs in either or both MM/PBSA and FlexddG scoring were picked. To further enhance the sequence diversity of the final candidates, three additional sequences located the closest to the centroid of sequence clusters other than clusters A and B in Fig. 3b were added to form a total of 23 final candidates from affinity maturation design for SPR binding assay. The sequence clusters were generated by the clustering algorithm HDBSCAN^87^ on a sequence space constructed by the first two principal components of 66 residue descriptors calculated by aaDescriptors^88^ on each residue position of the non-redundant Nb01 sequence variants.

### Statistics and Reproducibility

Independent experiments (*n* = 3) were performed during the SPR binding analysis between ELMO1-RBD (wild-type and mutants) and RhoG, and the competitive binding assay between Nb29 and RhoG binding to ELMO1-RBD, with the respective K_D_ and relative binding values expressed as means ± s.d. SPR kinetics of Nb01 and Nb29 binding to ELMO1-RBD was repeated by independent experiments (*n* = 2). Independent MD simulations (started from solvation until the end of production run) for the CDR loops flexibility assessment were repeated (*n* = 5). One-tailed, independent Student’s *t*-test was used for statistical testing between the RMSD of CDR2 and CDR3 between Nb01_apo_ and Nb29_apo_.

### Reporting summary

Further information on research design is available in the Nature Portfolio Reporting Summary linked to this article.

## Supplementary information

Supplementary Information

Description of Additional Supplementary Files

Supplementary Data 1

Supplementary Data 2

Reporting Summary

## Data Availability

The solution structure of ELMO1-RBD and the crystal structure of the ELMO1-RBD and RhoG complex have been deposited in the Protein Data Bank with IDs 6JPP and 7Y4A, respectively. The chemical shift assignments have been deposited in the Biological Magnetic Resonance Data Bank (BMRB) with accession code 36244. The source data behind the graphs in the paper are provided as Supplementary Data 1. The input and output files for the 10 ns MD production run shown in Fig. 3g are provided as Supplementary Data 2.

## Code availability

NMRPipe (v. 9.2), PatchDock (v. 1.3) and RosettaDock (v. 3.9) were used for data collection. CYANA (v. 2.1), AMBER 14, Coot (v. 0.9.4), Phenix (v. 1.14), PatchDock (v. 1.3) RosettaScripts (v. 3.9), GROMACS (v. 5.0.4) and FlexddG (v. 3.9) were used for data analysis. Python (v. 3.8.10) and packages *pandas* (v. 1.3.5), *MDAnalysis* (v. 2.4.2), *matplotlib* (v. 3.2.2), *scipy* (v. 1.7.3), *seaborn* (v. 0.11.2) and *HDBSCAN* (v. 0.8.1) were used for data processing and plotting of Fig. 3b, g and Supplementary Figure 6. GraphPad Prism (v. 9.0) was used for plotting Figs. 2c, 3a, b and e. Codes used for data processing and analysis are available at https://github.com/johnnytam100/elmo1-rhog_nanobody_design.
