# Supplementary material for: Targeting Ras-binding domain of ELMO1 by computational nanobody design
Source: Commun Biol. 2023 Mar 17;6:284. doi: 10.1038/s42003-023-04657-w (PMC10023680; doi:10.1038/s42003-023-04657-w)
Supplement: Supplementary file 1 — Supplementary Information [file 42003_2023_4657_MOESM1_ESM.pdf]

Supplementary Information

Targeting Ras-binding domain of ELMO1 by computational nanobody design

Chunlai Tam[1,2], Mutsuko Kukimoto-Niino[3,*], Yukako Miyata-Yabuki[4], Kengo Tsuda[3], Chiemi Mishima-Tsumagari[3], Kentaro Ihara[3], Mio Inoue[3], Mayumi Yonemochi[4], Kazuharu Hanada[3], Takehisa Matsumoto[4], Mikako Shirouzu[3,4], Kam Y. J. Zhang[1,2,*]

[1]Laboratory for Structural Bioinformatics, Center for Biosystems Dynamics Research, RIKEN, 1-7-22 Suehiro, Tsurumi, Yokohama, Kanagawa 230-0045, Japan
[2]Department of Computational Biology and Medical Sciences, Graduate School of Frontier Sciences, The University of Tokyo, Kashiwa, Chiba 277-8561, Japan
[3]Laboratory for Protein Functional and Structural Biology, Center for Biosystems Dynamics Research, RIKEN, 1-7-22 Suehiro, Tsurumi, Yokohama, Kanagawa 230-0045, Japan
[4]Drug Discovery Structural Biology Platform Unit, Center for Biosystems Dynamics Research, RIKEN, 1-7-22 Suehiro, Tsurumi, Yokohama, Kanagawa 230-0045, Japan

*Correspondence should be addressed to Mutsuko Kukimoto-Niino (email: kukimoto@riken.jp); Kam Y. J. Zhang (email: kamzhang@riken.jp)

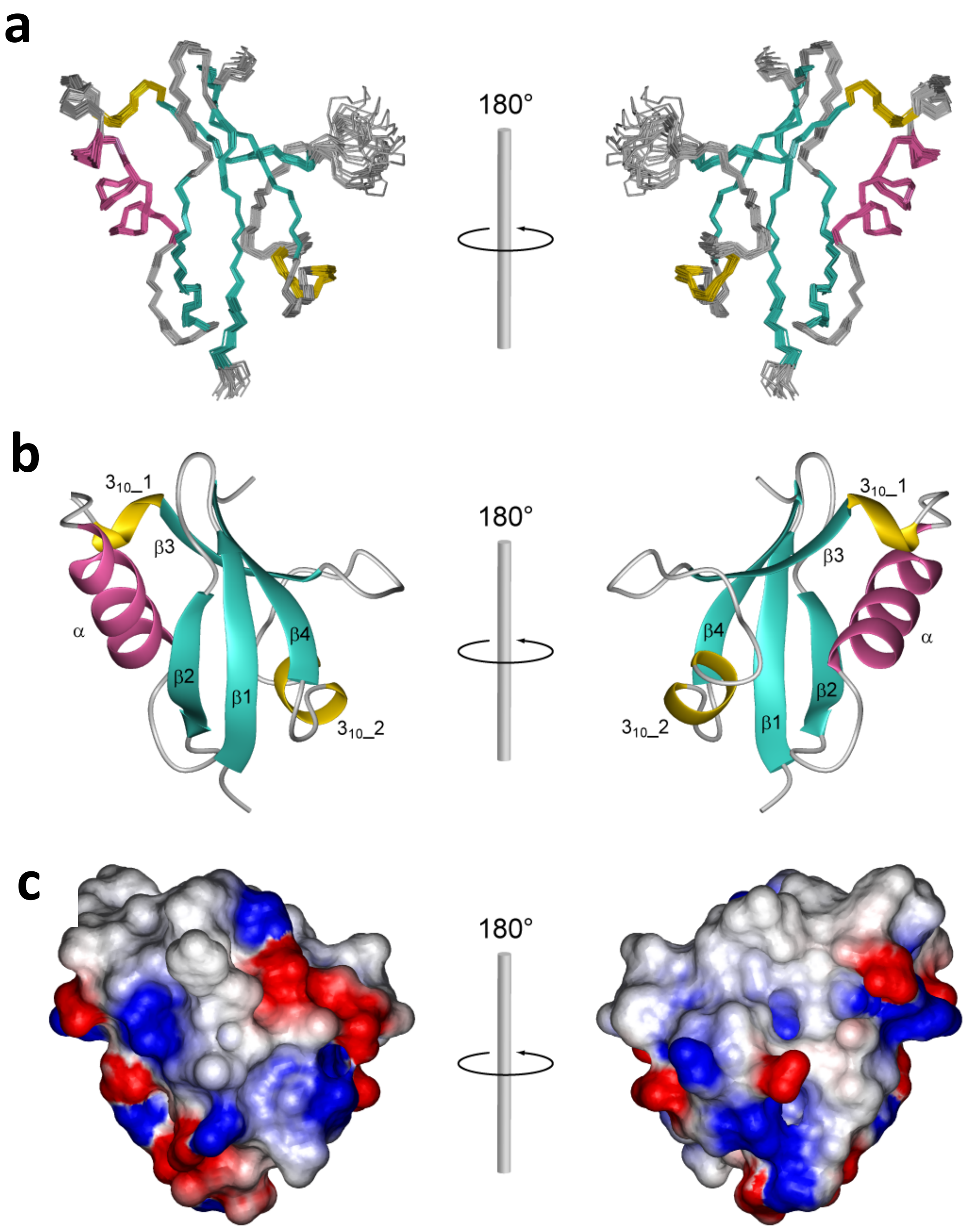

Supplementary Figure 1. Solution structure of ELMO1-RBD. **a** The backbone superimposition of the final 20 structures by NMR. **b** Ribbon representations of ELMO1 RBD with β-strands in cyan, α-helix in pink, and 3_{10}-helix in yellow. **c** Electrostatic surface representations of ELMO1-RBD. The protein surface is colored according to its electrostatic potential from red (negatively charged) to blue (positively charged).

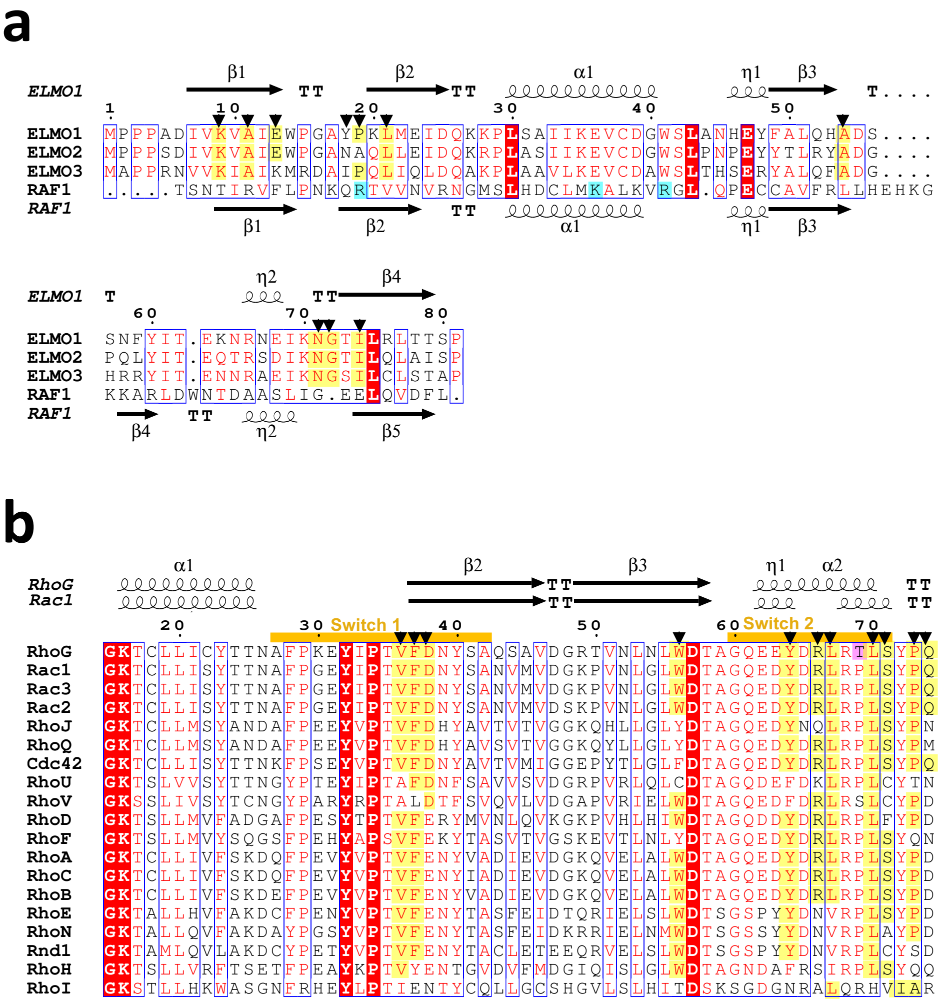

Supplementary Figure 2. Sequence alignments. **a** Sequence alignment of RBDs of human ELMO1-3 and a conventional Ras effector, Raf (RAF1). Secondary structures of ELMO1-RBD in complex with RhoG (this study) and Raf-RBD in complex with Ras (PDB ID: 4G0N) are indicated on top and bottom of the sequences, respectively. RhoG-binding residues in ELMO1 are indicated by arrowheads, of which the conserved residues are shown in yellow. To highlight the different GTPase binding mode between ELMO-RBD and Raf-RBD, clusters of basic residues of Raf that interact with Ras are shown in cyan.

b Sequence alignment of human Rho family GTPases. Switch 1 and 2 regions are shown in orange. Secondary structures of RhoG (this study) and Rac1 (PDB ID: 1E96) are indicated on top of the sequences. ELMO1-binding residues in RhoG are indicated by arrowheads, of which the conserved residues are shown in yellow. In Rac1-3, the closest isoforms of RhoG, all residues for binding to ELMO1 are conserved, but in switch 2 the secondary structure is different due to the difference in amino acid, Pro69 (helix breaker) in Rac and Thr69 in RhoG (shown in magenta).

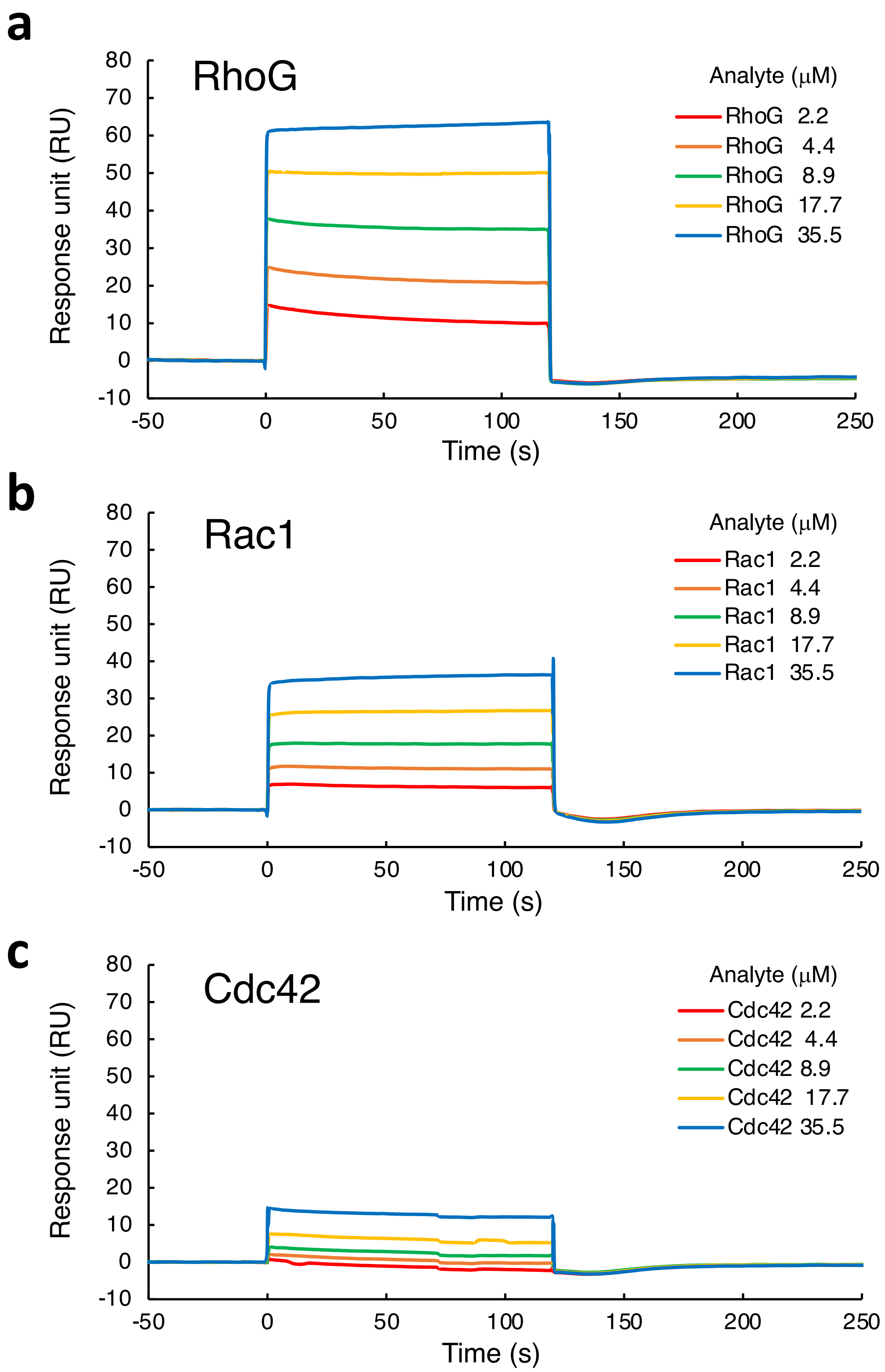

Supplementary Figure 3. SPR sensorgrams of five different concentrations of the GTPgS-bound **a** RhoG, **b** Rac1, and **c** Cdc42 against the GST-captured ELMO1-RBD.

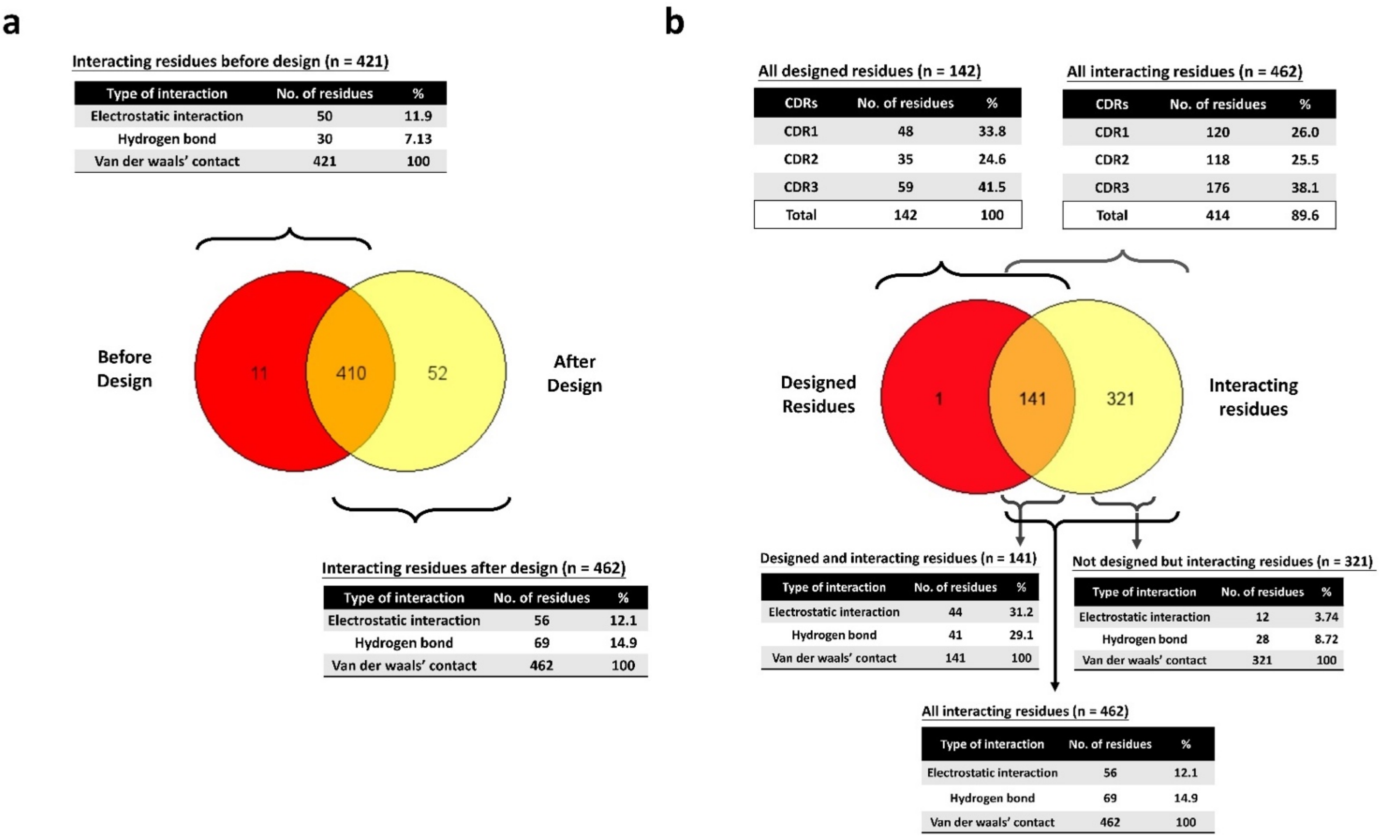

Supplementary Figure 4. Comparison of **a** change of types of interaction before and after design and **b** distribution of the designed and interacting residues on the three CDR loops and the types of interaction.

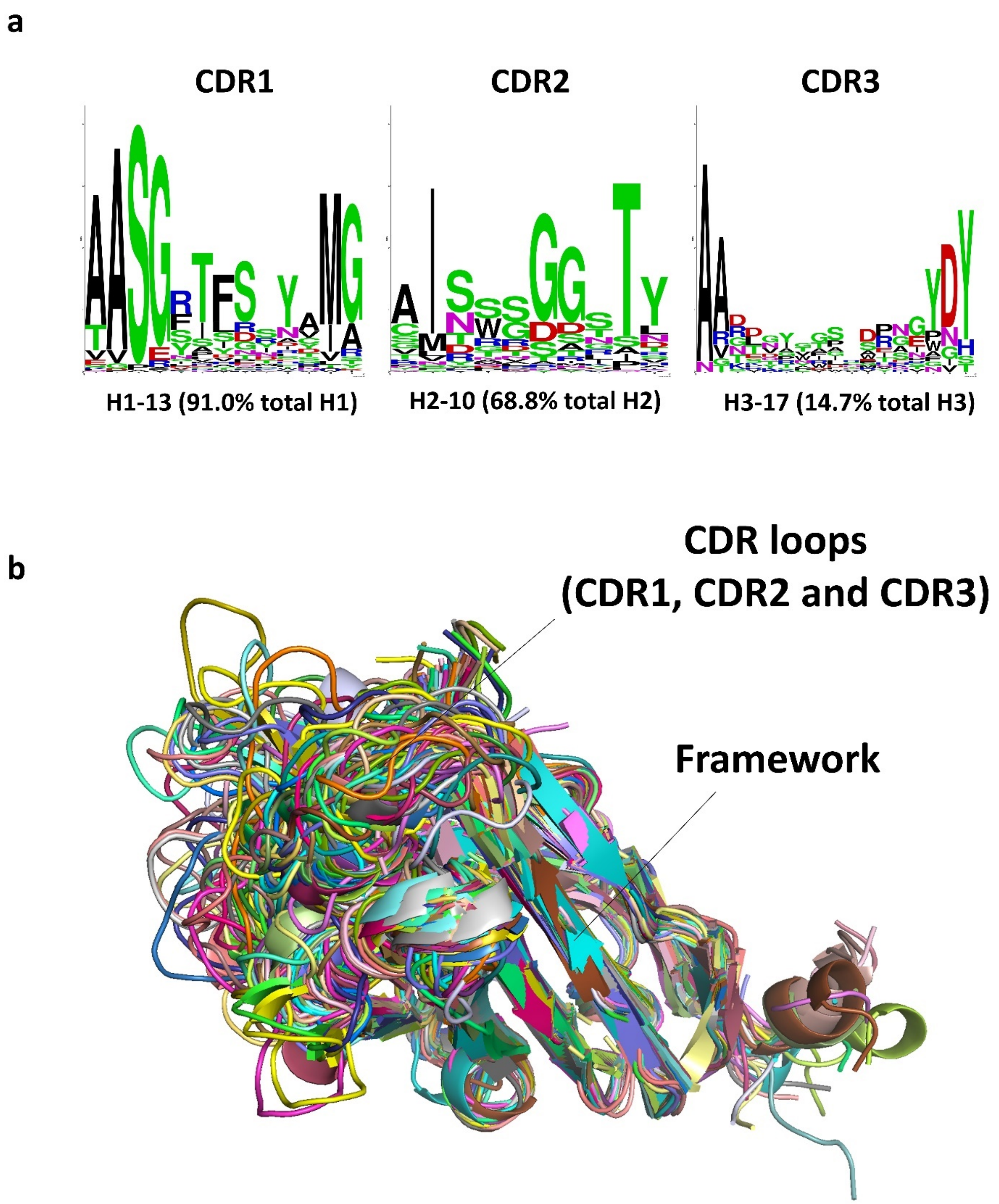

Supplementary Figure 5. **a** Sequence and **b** conformational diversity of CDR loops of the parent nanobodies. Sequence logos are showing the most abundant CDR sequence cluster from PyIgClassify for each CDR loop.

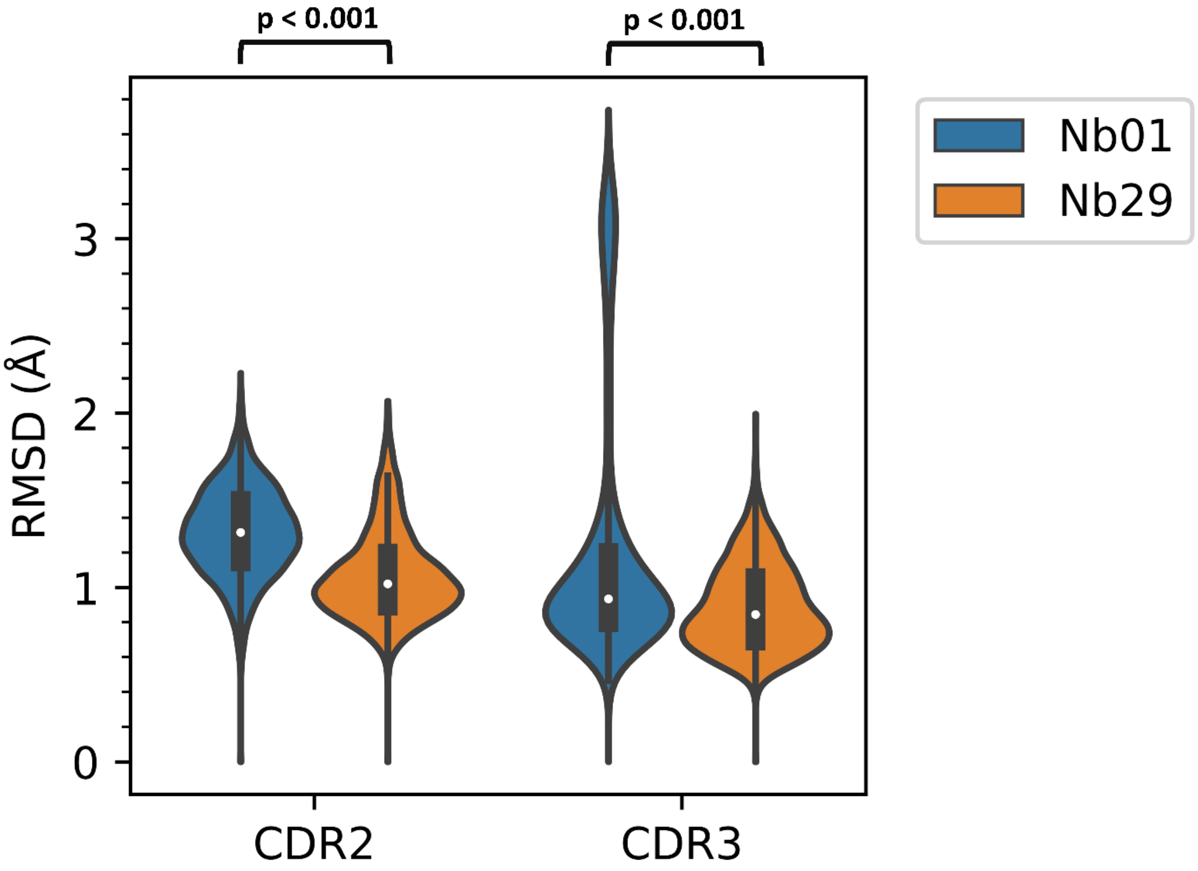

Supplementary Figure 6. Comparison of RMSD distributions of backbones of CDR2 and CDR3 between snapshots sampled from all 5-fold repeated, independent MD simulations against the input structures $Nb01_{apo}$ and $Nb29_{apo}$ as references. One-tailed, independent t-test was used for p-value calculation.

Supplementary Table 1. First-round design final selection scheme.

Criteria Sets	MM/PBSA	Funnel visual score (min. = 1, max. = 10)	Min. ddg-binding score	Number of mutations	Number of redundant sequences	SASA
1	negative	Min. 6				
2	lowest 20					
3		Min. 7				
4			lowest 20			
5	negative	Min. 5		Max. 8		
6	lowest 20			Max. 8		
7		Min. 7		Max. 8		
8			lowest 20	Max. 8		
9	negative				Min. 8	
10		Min. 7			Min. 8	
11			lowest 20		Min. 8	
12						highest 20

Supplementary Table 2. Affinity maturation design final selection scheme.

Criteria set	MM/PBSA	FlexddG	Number of designs selected
**1**	Lowest 50%	Lowest 50%	10
**2**	Lowest 5	-	5
**3**	-	Lowest 5	5

Supplementary Note 1

Pulse program of 2D ^{13}C-HSQC using State-TPPI and adiabatic pulses for inversion

The pulse program of 2D ^{13}C-HSQC was created to (i) reduce the effect of large ^{13}C-^{13}C coupling constants (especially aromatic rings) and (ii) efficiently suppress the signal due to water, as follows:

```
;kt_hsqcphprgpsppp
;hsqcphpr modified for the 13C/15N-labeled sample dissolved in H2O buffer.
;               by K. Tsuda and M. Oouchi (2018/1/17) Riken
;avance III version [Topspin 3.2]
;13C-HSQC
;2D H-1/C correlation via double inept transfer
;phase sensitive (t1)
;with 15N refocussing in t1 evolution
;with decoupling during acquisition
;with presaturation (pl9) during relaxation delay followed by gradient pulse
;using purge pulses before d1
;using shaped pulse for inversion (sp13) on f2 - channel
;(use parameterset HSQCETGPSP.3_ADIA)
;
;$CLASS=HighRes
;$DIM=2D
;$TYPE=
;$SUBTYPE=
;$COMMENT=

prosol relations=<triple>

#include <Avance.incl>
#include <Grad.incl>
#include <Delay.incl>


"in0=inf1/2"


"p2=p1*2"
```

```
"p22=p21*2"


"d4=1s/(cnst2*4)"
"d11=30m"
"d12=20u"

"d0=in0-p3*2/PI-larger(p22,p2)/2"       ; F1(180, -360)
;-----------------------------------------
"DELTA1=d4-larger(p2,p8)/2-p19-d16"
"DELTA2=d4-larger(p2,p8)/2-p19-d16-24u-de"
;-----------------------------------------


"cnst13=75"                             ; check!!
;- Offset freq for F2 shaped pulse
"spoff13=bf2*cnst13/1000000-o2"
;---------------------------------


"acqt0=0"
baseopt_echo


1 ze
   d11 pl12:f2
2 d11 do:f2

3 d12 pl10:f1                                        ; For purge pulses (p17, pl10)
   p17 ph1
   p17*2 ph2

4 d12 pl9:f1                            ; For presaturation (pl9)
   d1 cw:f1 ph29
   4u do:f1
   d12 pl1:f1

   50u UNBLKGRAD
```

```
   p16:gp3                         ; Gradient 3 [4%]
   d16 pl0:f2

;--------------- HSQC ---------------
   (p1 ph1)
   DELTA1
   p19:gp4                         ; Gradient 4 [15%]
   d16 pl0:f2
   (center (p2 ph1) (p8:sp13 ph1):f2)   ; modified to shaped pulse (sp13).
   DELTA1
   p19:gp4                         ; Gradient 4 [15%]
   d16 pl2:f2
   (p1 ph2)
   4u
   p16:gp1                         ; Gradient 1 [40%]
   d16

;*** t1(13C) evolution ************
   (p3 ph3):f2                         ; phase!

   d0
   (center (p2 ph1) (p22 ph1):f3)              ; modified. 1H and 15N refocussing. F1(180,-360)
   d0

   (p3 ph4):f2                         ; ph4!!
;**********************************

   4u
;    p16:gp1*0.2514                    ; Gradient 1 [40%]*[Gr(13C)/Gr(1H)]
   p16:gp2                         ; Gradient 2 [10%]
   d16
   (p1 ph1)
   DELTA1
   p19:gp4*-1                         ; Gradient 4 [15%]*-1
   d16 pl0:f2
   (center (p2 ph1) (p8:sp13 ph1):f2)   ; modified to shaped pulse (sp13).
   d16
```

```
   p19:gp4*-1                       ; Gradient 4 [15%]*-1
   DELTA2 pl12:f2
;   DELTA1 pl12:f2
;-----------------------------------
;   (p1 ph1)                        ; residual H2O resonances to the z axis

   4u BLKGRAD
   20u
   go=2 ph31 cpd2:f2
   d11 do:f2 mc #0 to 2
        F1PH(calph(ph3, +90), caldel(d0, +in0))
exit


ph1=0
ph2=1
ph4=0 0 2 2
;- 13C phase for t1 evolution
ph3=0 2
;----------------------------
ph29=0
ph31=0 2 2 0


;pl0: 0W
;pl1: f1 channel - power level for pulse (default)
;pl2: f2 channel - power level for pulse (default)
;pl3: f3 channel - power level for pulse (default)
;pl9: f1 channel - power level for presaturation
;pl10: f1 channel - power level for TOCSY-spinlock         [default]
;pl12: f2 channel - power level for CPD/BB decoupling
;pl32: f1 channel - power level for low power presaturation
;sp13: f2 channel - shaped pulse 180 degree (adiabatic)
;spnam13: Crp80,0.5,20.1 (p8)
;p1: f1 channel -   90 degree high power pulse
;p2: f1 channel - 180 degree high power pulse
;p3: f2 channel -   90 degree high power pulse
;p8: f2 channel - 180 degree shaped pulse for inversion (sp13)
```

```
;p16: homospoil/gradient pulse        [1 msec]
;p17: f1 channel - trim pulse        [2.5 msec]
;p19: homospoil/gradient pulse 2        [600 usec]
;p21: f3 channel - 90 degree high power pulse
;p22: f3 channel - 180 degree high power pulse
;d0: incremented delay (2D)        [in0-p3*2/PI-larger(p22,p2)/2]
;d1: relaxation delay; 1-5 * T1
;d4: 1/(4 * J(CH)av)
;d11: delay for disk I/O        [30 msec]
;d12: delay for power switching        [20 usec]
;d16: delay for homospoil/gradient recovery
;cnst2: = J(CH)av        [145 (or 170 <120~220>) Hz]
;cnst13: Offset frequency for f2 channel shaped pulse        [75 ppm]
;inf1: 1/SW(C) = 2 * DW(C)
;in0: 1/(2 * SW(C)) = DW(C)
;nd0: 2
;ns: 4 * n
;ds: >= 16
;td1: number of experiments
;FnMODE: States-TPPI, TPPI, States or QSEQ
;cpd2: decoupling according to sequence defined by cpdprg2
;                       bi_p5m4sp_4sp.2


;use gradient ratio:      gp 1: gp 2: gp 3: gp 4
;                                 40:    10:     4:    15

;for z-only gradients:
;gpz1: 40% (p16)
;gpz2: 10% (p16) <+-0.01~>
;gpz3: 4% (p16) following presaturation
;gpz4: 15% (p19)

;use gradient files:
;gpnam1: SMSQ1000
;gpnam2: SMSQ1000
;gpnam3: SMSQ1000
;gpnam4: SMSQ1000
```

;Processing

;PHC0(F1): 180
;PHC1(F1): -360
;FCOR(F1): 1

;$Id: hsqcphpr,v 1.5.8.1 2012/01/31 17:56:32 ber Exp $

Supplementary Note 2

Pulse program of 3D ^{13}C-NOESY-HSQC using State-TPPI and adiabatic pulses for inversion

The pulse program of 3D ^{13}C-NOESY-HSQC was created to (i) reduce the effect of large ^{13}C-^{13}C coupling constants (especially aromatic rings), (ii) efficiently suppress the signal due to water, and (iii) purge zero quantum coherences (ZQCs) by pulse gradients, as follows:

```
;kt_noesyhsqcphprgpsppp3d
;hsqcphpr modified for the 13C/15N-labeled sample dissolved in H2O buffer.
;               by K. Tsuda and M. Oouchi (2018/1/17) Riken
;avance III version [Topspin 3.2]
;13C-NOESY-HSQC
;3D sequence with
; homonuclear correlation via dipolar coupling
; dipolar coupling may be due to noe or chemical exchange
; H-13/C correlation via double inept transfer
;phase sensitive (t1)
;phase sensitive (t2)
;with gradient pulses in mixing time (d8)
;with 15N refocussing in t1 and t2 evolutions
;with decoupling during acquisition
;with presaturation (pl9) during relaxation delay followed by gradient pulse
;using purge pulses before d1
;using shaped pulse for inversion (sp13) on f2 - channel
;(use parameterset NOESYHSQCETGP3D)
;
;$CLASS=HighRes
;$DIM=3D
;$TYPE=
;$SUBTYPE=
;$COMMENT=

prosol relations=<triple>

#include <Avance.incl>
#include <Grad.incl>
#include <Delay.incl>
```

```
"in0=inf1/2"
"in10=inf2/2"


"p2=p1*2"
"p22=p21*2"


"d4=1s/(cnst2*4)"
"d11=30m"
"d12=20u"

;"d0=2u"
"d0=in0/2-p1*2/PI"                    ; F1( 90, -180)
#     ifdef LABEL_CN
"d10=in10-p3*2/PI-larger(p22,p2)/2"   ; F2(180, -360)
#     else
"d10=in10-p3*2/PI-p2/2"     ; F2(180, -360)
#     endif /*LABEL_CN*/
;-----------------------------------------
"DELTA1=d4-larger(p2,p8)/2-p19-d16"
;"DELTA2=d4-larger(p2,p8)/2-p19-d16-4u-de"
"DELTA2=d4-larger(p2,p8)/2-p19-d16-4u"

"TAU=d8/2-p16*2-d16-p2/2"
;-----------------------------------------


"cnst13=75"                           ; check!!
;- Offset freq for F2 shaped pulse
"spoff13=bf2*cnst13/1000000-o2"
;---------------------------------


"acqt0=0"
baseopt_echo
```

```
aqseq 321


1 ze
   d11 pl12:f2
2 d11 do:f2
   20u BLKGRAD
3 d12 pl10:f1                                   ; For purge pulses (p17, pl10)
   p17 ph1
   p17*2 ph2

4 d12 pl9:f1                           ; For presaturation (pl9)
   d1 cw:f1 ph29
   4u do:f1
   d12 pl1:f1

   50u UNBLKGRAD

   p16:gp3                                        ; Gradient 3 [4%]
   d16

;--------------- NOE ----------------
;*** t1(1H) evolution *************
#    ifdef LABEL_CN
   (
    center (p1 ph7 d0 d0 p1 ph1):f1    ; phase!
    (p8:sp13 ph1):f2
    (p22 ph1):f3
   )
#    else
   (
    center (p1 ph7 d0 d0 p1 ph1):f1    ; phase!
    (p8:sp13 ph1):f2
   )
#    endif /*LABEL_CN*/
```

```
;**********************************

   TAU
   p16*2:gp5                          ; Gradient 5 [20%] in mixing time
   d16
   (p2 ph1):f1
   d16
   p16*2:gp5*-1                       ; Gradient 5 [20%]*-1 in mixing time
   TAU

;--------------- HSQC ---------------
   (p1 ph1)
   DELTA1
   p19:gp4                      ; Gradient 4 [15%]
   d16
   (center (p2 ph1) (p8:sp13 ph1):f2)   ; modified to shaped pulse (sp13).
   DELTA1
   p19:gp4                      ; Gradient 4 [15%]
   d16 pl2:f2
   (p1 ph2)
   4u
   p16:gp1                      ; Gradient 1 [40%]
   d16

;*** t2(13C) evolution ************
   (p3 ph3):f2                        ; phase!

   d10

#    ifdef LABEL_CN
   (center (p2 ph1) (p22 ph1):f3)             ; modified. 1H and 15N refocussing. F2(180,-360)
#    else
   (p2 ph1)
#    endif /*LABEL_CN*/

   d10

   (p3 ph4):f2                        ; ph4!!
```

```
;*********************************

   4u
;   p16:gp1*0.2514                              ; Gradient 1 [40%]*[Gr(13C)/Gr(1H)]
   p16:gp2                  ; Gradient 2 [10%]
   d16
   (p1 ph1)
   DELTA1
   p19:gp4*-1                    ; Gradient 4 [15%]*-1
   d16
   (center (p2 ph1) (p8:sp13 ph1):f2)   ; modified to shaped pulse (sp13).
   d16
   p19:gp4*-1                    ; Gradient 4 [15%]*-1
   DELTA2 pl12:f2
;   DELTA1 pl12:f2
;-----------------------------------
;   (p1 ph1)                                    ; residual H2O resonances to the z axis

   4u
   go=2 ph31 cpd2:f2
   d11 do:f2 mc #0 to 2
        F1PH(calph(ph7, +90), caldel(d0, +in0))
        F2PH(calph(ph3, +90), caldel(d10, +in10))

   20u BLKGRAD

exit



ph1=0
ph2=1
ph4=0 0 0 0 2 2 2 2
;- 13C phase for t2 evolution
ph3=0 2
;- 1H phase for t1 evolution
ph7=0 0 2 2
;-----------------------------
ph29=0
```

```
ph31=0 2 2 0 2 0 0 2


;pl0: 0W
;pl1: f1 channel - power level for pulse (default)
;pl2: f2 channel - power level for pulse (default)
;pl3: f3 channel - power level for pulse (default)
;pl9: f1 channel - power level for presaturation
;pl10: f1 channel - power level for TOCSY-spinlock         [default]
;pl12: f2 channel - power level for CPD/BB decoupling
;pl32: f1 channel - power level for low power presaturation
;sp13: f2 channel - shaped pulse 180 degree (adiabatic)
;spnam13: Crp80,0.5,20.1 (p8)
;p1: f1 channel -  90 degree high power pulse
;p2: f1 channel - 180 degree high power pulse
;p3: f2 channel -  90 degree high power pulse
;p8: f2 channel - 180 degree shaped pulse for inversion (sp13)
;p16: homospoil/gradient pulse         [1 msec]
;p17: f1 channel - trim pulse         [2.5 msec]
;p19: homospoil/gradient pulse 2         [600 usec]
;p21: f3 channel - 90 degree high power pulse
;p22: f3 channel - 180 degree high power pulse
;d0: incremented delay (F1 in 3D)         [in0/2-p1*2/PI]
;d1: relaxation delay; 1-5 * T1
;d4: 1/(4 * J(CH)av)
;d8: mixing time         [intra: 80 msec/inter: 150 msec]
;d10: incremented delay (F2 in 3D)         [in10-p3*2/PI-larger(p22,p2)/2]
;d11: delay for disk I/O         [30 msec]
;d12: delay for power switching         [20 usec]
;d16: delay for homospoil/gradient recovery
;cnst2: = J(CH)av         [145 (or 170 <120~220>) Hz]
;cnst13: Offset frequency for f2 channel shaped pulse         [75 ppm]
;inf1: 1/SW(H) = 2 * DW(H)
;inf2: 1/SW(C) = 2 * DW(C)
;in0: 1/(2 * SW(H)) =  DW(H)
;nd0: 2
;in10: 1/(2 * SW(C)) = DW(C)
;nd10: 2
```

```
;ns: 8 * n
;ds: >= 16
;td1: number of experiments in F1
;td2: number of experiments in F2
;FnMODE: States-TPPI (or TPPI) in F1
;FnMODE: States-TPPI (or TPPI) in F2
;cpd2: decoupling according to sequence defined by cpdprg2
;                    bi_p5m4sp_4sp.2


;use gradient ratio:    gp 1: gp 2: gp 3: gp 4: gp 5
;                        40:   10:    4:   15:   20

;for z-only gradients:
;gpz1: 40% (p16)
;gpz2: 10% (p16) <+-0.01~>
;gpz3: 4% (p16) following presaturation
;gpz4: 15% (p19)
;gpz5: 18% (p16*2) in mixing time

;use gradient files:
;gpnam1: SMSQ10.100
;gpnam2: SMSQ10.100
;gpnam3: SMSQ10.100
;gpnam4: SMSQ10.100
;gpnam5: SMSQ10.100


;Processing

;PHC0(F1): 90
;PHC1(F1): -180
;FCOR(F1): 1

;PHC0(F2): 180
;PHC1(F2): -360
;FCOR(F2): 1
```

;$Id: hsqcphpr,v 1.5.8.1 2012/01/31 17:56:32 ber Exp $
