# Supplementary material for: Targeting Ras-binding domain of ELMO1 by computational nanobody design
Source: Commun Biol. 2023 Mar 17;6:284. doi: 10.1038/s42003-023-04657-w (PMC10023680; doi:10.1038/s42003-023-04657-w)
Supplement: Supplementary file 2 — Description of Additional Supplementary Files [file 42003_2023_4657_MOESM2_ESM.pdf]

Description of Additional Supplementary Files

File name: Supplementary Data 1
Description: The source data behind the graphs in the paper.

File name: Supplementary Data 2
Description: Input and output files for the 10 ns MD production run shown in Fig. 3G.
